# Case Report: Neoadjuvant immunotherapy with pembrolizumab alone for bilateral upper tract urothelial carcinoma is a feasible strategy for kidney sparing and avoidance of hemodialysis

**DOI:** 10.3389/fonc.2022.985177

**Published:** 2022-09-23

**Authors:** Wai-Nga Chan, Yun-Ching Huang, Dong-Ru Ho, Chih-Shou Chen

**Affiliations:** ^1^Divisions of Urology, Department of Surgery, Chang Gung Medical Foundation, Chiayi City, Taiwan; ^2^Department of Medicine, College of Medicine, Chang Gung University, Taoyuan, Taiwan

**Keywords:** case report, upper tract urothelial carcinoma, immune checkpoint inhibitors, neoadjuvant immunotherapy, kidney sparing

## Abstract

In Taiwan, the incidence of upper-tract urothelial carcinomas (UTUCs) is higher than in western countries (20%–31% vs. 5%–10%), as is bilateral disease. The standard management for high-grade UTUC is radical nephroureterectomy with bladder cuff excision and regional lymphadenectomy. The challenges in managing bilateral UTUCs are how to retain renal function and avoid permanent hemodialysis. We present two cases of developed bilateral high-grade renal pelvis urothelial carcinoma, cT3N0M0 stage III, that revealed excellent results in tumor regression after three cycles of half-dose pembrolizumab. One case received unilateral retroperitoneal laparoscopic nephroureterectomy with bladder cuff excision; thereafter, renal function has been good until now, and the remaining right kidney has been free of tumor recurrence in the 3 years of follow-up. The other patient, however, expired from an immune-related adverse event (irAE) 22 days after the third cycle of pembrolizumab, although tumor remission was evident also. Neoadjuvant pembrolizumab alone could be a potential strategy in positive of selected biomarkers for high-grade bilateral UTUC with remaining neglectable nephrotoxicity and may avoid permanent hemodialysis.

## Introduction

In Taiwan, the incidence of upper tract urothelial carcinomas (UTUCs) is higher than in western countries (20%–31% vs. 5%–10%) (1–7); moreover, most UTUCs are presented unilaterally. The incidence of synchronous or metachronous bilateral disease is about 1.6% to 6.0% in western countries but is 13% of the general population in Taiwan (8–12). The challenges in managing bilateral UTUCs are how to retain renal function and avoid permanent hemodialysis. Nowadays, immune checkpoint inhibitors indicating neglectable nephrotoxicity have been one of the alternative approaches used in treating advanced or metastatic bladder urothelial carcinoma (UC), such as in the PURE-01 study (13), although single pembrolizumab neoadjuvant therapy PURE-02 seems to not provide a promising result in biomarker-unselected high-risk localized UTUCs (14).

## Case description

### Case 1

**Figure 1:**
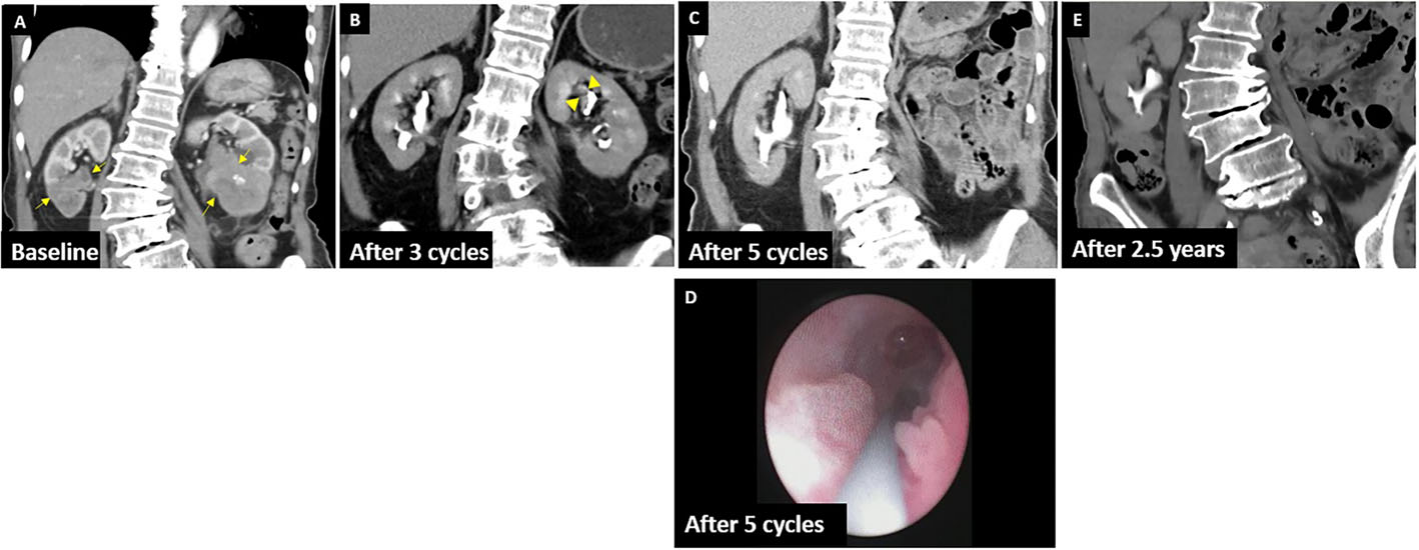
CTU of case 1. **(A)** Baseline: arrows show the UC in the bilateral renal pelvis with parenchyma invasion. **(B)** After three cycles: arrowheads show a residual tumor in the left upper calyx. **(C)** CTU revealed no recurrence after five cycles. **(D)** Showing a suspected lesion at the right middle ureter in URS with dysplasia in the pathology report. **(E)** CTU revealed no recurrence in the latest follow-up (January 2021).

An 81-year-old woman with a history of hypertension and Alzheimer’s disease had presented with intermittent painless gross hematuria for 1 year. She had not consumed alcohol nor smoked cigarettes, and there was no cancer history in her family. Her computed tomography (CT) of the abdomen revealed a tumor in the bilateral renal pelvis with renal parenchyma invasion (**Figure 1A**), and the pathology report from retrograde ureterorenoscopy biopsy showed high-grade invasive urothelial carcinoma (UC), while immunohistochemistry analysis revealed a high expression of programmed death-ligand-1 (PD-L1; Dako 22C3 pharmDx assay, Agilent Technologies, Santa Clara, California, USA) with a tumor proportion score of 60% in July 2018. We prescribed 100 mg of pembrolizumab every 3 weeks for three cycles for this locally advanced (cT3N0M0, stage III) bilateral renal pelvis high-grade UC with normal renal function. She did not have any severe adverse events during immunotherapy. Clinical characteristics during immunotherapies are shown in **Table 1**. Thereafter, her CT urography (CTU) showed a complete response of the right renal pelvis UC but a partial response of the left renal pelvis UC (**Figure 1B**). Therefore, she received a left retroperitoneal laparoscopic nephroureterectomy with bladder cuff excision in October 2018. The pathology report revealed residual invasive high-grade papillary UC, ypT3Nx.

**Table 1:** Clinical characteristics of case 1.

Variable	First cycle	Second cycle	Third cycle
Weight (kg)	43.4	41.7	42.7
Height (cm)	141	141	141
Body surface area	1.3	1.28	1.29
Hb (g/dl)	10.5	10.5	10.5
Creatinine (mg/dl)	0.96	0.97	0.87
AST (U/L)	23	24	24
ALT (U/L)	–	14	–
TSH (uIU/ml)	2.2	0.9	1.9
T4 (µg/dl)	7.4	8.7	7.0

AST, aspartate aminotransferase; ALT, alanine transaminase; TSH, thyroid-stimulating hormone (range: 0.27–4.2 uIU/ml); T4, thyroxine (range: 4.8–12.5 µg/dl).

We continued administering an additional two cycles of pembrolizumab 5 weeks after the operation, with an interval of 3 weeks because of ypT3N0M0. CTU was followed up 5 weeks after the last immunotherapy, which revealed negative local recurrence (**Figure 1C**). Right ureterorenoscopy (URS) showed a suspected lesion in the middle ureter with a pathology report indicating dysplasia in January 2019 (**Figure 1D**). Surveillance by CT images, urine cytology, and cystoscopy every 6 months was suggested, and all negative results of recurrence, including the previous suspicious dysplasia site, were reported even in the latest CTU in January 2021 (**Figure 1E**).

The patient was followed up in the outpatient department for 3 years. Her last creatinine level measurement was 1.12 mg/dl in January 2021 as she was lost to follow-up at the urological clinic due to the progression of dementia, although the neurological department had no mention of hematuria in their follow-up.

### Case 2

**Figure 2:**
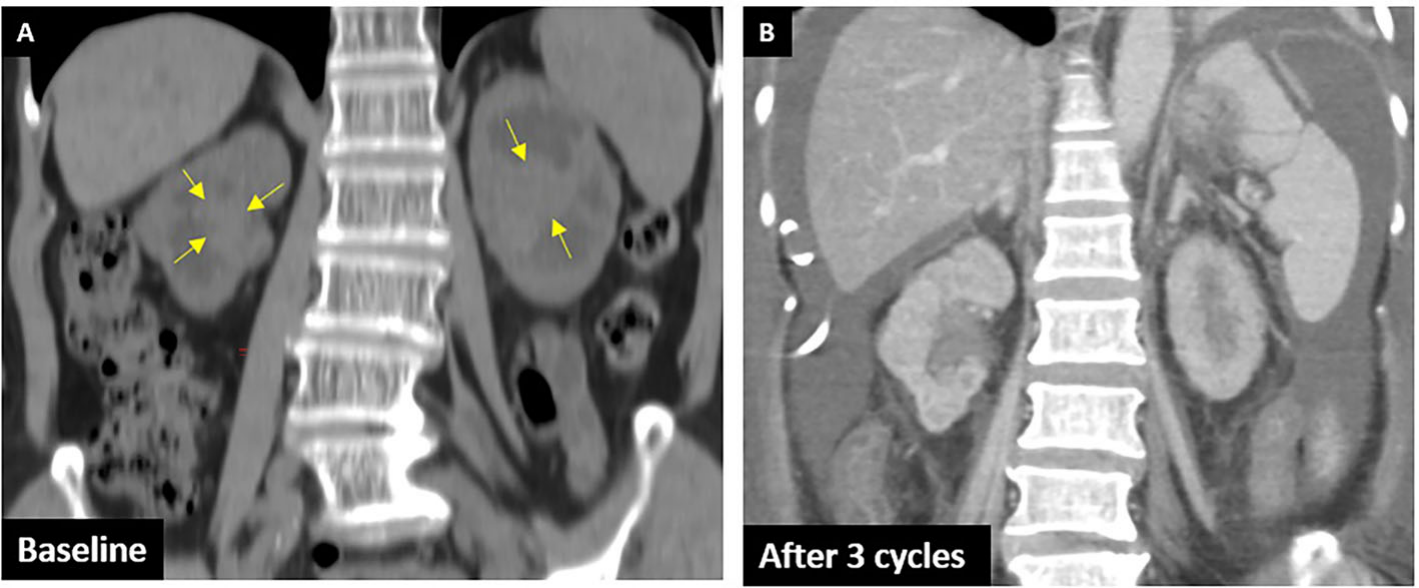
CTU of case 2. **(A)** Baseline: arrows show the UC in the bilateral renal pelvis with parenchyma invasion. **(B)** After three cycles: shows remission of bilateral tumors.

A 68-year-old woman with a history of type II diabetes and chronic kidney disease was found to have elevated creatinine from 2.3 to 4.86 mg/dl and microscopic hematuria for several months. She did not consume alcohol or smoke cigarettes, and there was no cancer history in her family. Her noncontrast CT of the abdomen revealed a tumor in the bilateral renal pelvis with renal parenchymal invasion and a right ureteral tumor (**Figure 2A**). URS showed a polypoid tumor with intraluminal occlusion in the right ureter and an infiltrative tumor in the left. The pathology report of the biopsy revealed high-grade UC, and the immunohistochemistry analysis showed expression of PD-L1 (Dako 22C3 pharmDx assay) with a Combined Positive Score (CPS) of 10 in October 2019. We dispensed three cycles of 100 mg of pembrolizumab every 3 weeks for locally advanced bilateral renal pelvis and right ureteral high-grade UC, cT3N0M0 stage III. Clinical characteristics during these immunotherapies are shown in **Table 2**. CT of the abdomen showed regression of bilateral renal pelvis tumors thereafter (**Figure 2B**).

**Table 2:** Clinical characteristics of case 2.

Variable	First cycle	Second cycle	Third cycle
Weight (kg)	46	45	46
Height (cm)	145	145	145
Body surface area	1.36	1.35	1.36
Hb (g/dl)	10.1	9.0	10.4
Creatinine (mg/dl)	4.23	4.14	3.74
AST (U/L)	16	–	–
ALT (U/L)	–	–	–
TSH (uIU/ml)	1.10	–	–
Free T4 (ng/dl)	1.10	–	–

Free T4 (range: 0.93–1.7 ng/dl).

However, she visited the emergency department (ED) 22 days after the third cycle because of low abdominal pain for 2 days accompanied by decreased urine output and dysuria without fever, chills, tachypnea, hematuria, or diarrhea. She was found to have acute decompensated hepatitis, acute pancreatitis, and acute-on-chronic kidney disease. We prescribed methylprednisolone due to grade 3 immune-related adverse event (irAE) being highly suspected. Unfortunately, her condition deteriorated quickly; she developed sinus bradycardia and hypothyroidism within 29 days and eventually expired.

## Discussion

UTUCs are rare in western countries and make up only 5%–10% (1, 2). By contrast, a much higher incidence of 20%–31% of UTUCs in Taiwan has been reported, especially within arsenic-related endemic areas of Blackfoot disease in southern Taiwan (3, 4). The annual incidence rate has been estimated at 1.2–4.7 cases per 100,000 person-years globally (5, 6) as compared to 3.14–3.41 per 100,000 person-years in Taiwan (7). Most UTUCs are presented unilaterally, with the incidence of the synchronous or metachronous bilateral disease being about 1.6% to 6.0% in western countries (8–11). In Taiwan, however, Huang et al. have demonstrated a 13% incidence rate of synchronous or metachronous bilateral UTUCs (12).

The standard management for high-grade UTUC is radical nephroureterectomy with bladder cuff excision and regional lymphadenectomy (5). Our report of unilateral pT3N0M0 UTUC revealed postoperative adjuvant chemotherapy offers a 5-year-recurrence-free survival rate of 74.4% versus 54.4% in nonadjuvant chemotherapy (15). However, for our patients, bilateral radical nephroureterectomy will result in life-long hemodialysis. The alternative approach for such cases is neoadjuvant chemotherapy. A meta-analysis demonstrated that the pathologic complete response rate (pCR, ypT0N0M0) was 11% and the pathologic partial response rate (pPR, ≤ypT1N0M0) was 43% (16). Additionally, a phase II trial of neoadjuvant chemotherapy of accelerated methotrexate, vinblastine, doxorubicin, and cisplatin for high-grade UTUC demonstrated a pCR of 14% and 62% in pPR with a final pathologic stage of ≤ ypT1. Also, the grade 3–4 toxicity rate was 23% in the aMVAC arm (17). However, neoadjuvant chemotherapy was administrated to select patients who had adequate renal function and good ECOG performance but whose renal function would decline following nephroureterectomy and might thereafter preclude adjuvant chemotherapy.

Nowadays, an immune checkpoint inhibitor is one of the alternative approaches in advanced or metastatic bladder urothelial carcinoma for first-line systemic therapy in selective patients or second-line therapy. Numerous studies have investigated the efficacy and safety of immune checkpoint inhibitors (ICI) compared to chemotherapy; for example, Bellmunt et al. demonstrated longer median overall survival (OS, 10.3 vs. 7.4 months; *p* = 0.002) and fewer grade 3–5 treatment-related adverse events (AE) in pembrolizumab-treated patients compared to chemotherapy (15.0% vs. 49.4%) in recurring or progressing UC after platinum-based chemotherapy (18). Furthermore, a phase III KEYNOTE-361 trial that included 65 patients (21%) with UTUC in a pembrolizumab group analyzing first-line pembrolizumab compared to chemotherapy alone resulted in similar OS (14.3 vs. 15.6 months) as well as high PD-L1 expression by a CPS of at least 10 (16.1 vs. 15.2 months) in locally advanced or metastatic cisplatin-ineligible urothelial carcinoma (19).

Moreover, an open-label, single-arm, phase II PURE-01 study investigated the use of neoadjuvant pembrolizumab in muscle-invasive bladder carcinoma resulted in complete pathological remission (ypT0) in 42% of patients and downstaging (<ypT2) in 54% after receiving three cycles of 200 mg pembrolizumab before radical cystectomy, and also revealed better response rate in patients with PD-L1 CPS ≥10% and was safely administrated (13).

ICI has already changed our clinical approach in advanced or metastatic bladder UC. However, there is limited evidence of kidney sparing approaches in high-grade UTUC, probably because of rare incidence in western countries, with bilateral high-grade not even being mentioned. Since the experience in PURE-01 is promising and possesses lesser adverse events than chemotherapy, we expected the same rationale would be matched to avoid hemodialysis in bilateral UTUC patients.

We prescribed half-dosage (100 mg) pembrolizumab because the patients’ weights were 43 and 45 kg only. In our experience of these two cases that developed bilateral high-grade renal pelvis UC, both had an excellent result in tumor regression after three cycles, even at a half-dosage (100 mg) of pembrolizumab compared to the recommended dose. One patient had the right kidney preserved with complete remission and received a left nephroureterectomy with bladder cuff excision for the partially responding tumor and has avoided lifelong hemodialysis without recurrence in 3 years of follow-up until now. Accordingly, a patient with bilateral UTUC with positive selected biomarkers might benefit from neoadjuvant pembrolizumab as the only strategy to avoid lifelong hemodialysis.

Unfortunately, the other case expired from severe irAE even though having a great response to pembrolizumab; accordingly, there remains a risk of 15%–17% grade 3–5 treatment-related adverse events with pembrolizumab (18, 19). For patient safety, the main problem is early recognition and differential diagnosis followed by proper treatments. We suggest clinicians follow the laboratory studies regularly and develop an awareness of the diversity of presentations of irAE. Different specialists should pay more attention to early diagnosis and effective treatment in case these patients would visit the ED first. The three cycles of half-doses of neoadjuvant pembrolizumab when combined with an additional two booster cycles might offer promising results in bilateral UTUC with positive biomarker-selected patients expecting not to have lifelong hemodialysis.

## Conclusion

According to the existing evidence of ICI in treating advanced or metastatic UC and our experiences, neoadjuvant pembrolizumab alone could be a potential strategy in PD-L1 expression high-grade bilateral UTUC with remaining neglectable nephrotoxicity, is easy to use, and, in particular, could avoid lifelong hemodialysis. Additionally, there is no consensus on the booster of immunotherapy after radical procedures, although, in our limited experience, two booster cycles of pembrolizumab appeared to suffice in our patients. More prospective clinical trials and validations are needed to build up standard management and biomarkers for UTUC to offer more benefits in bilateral disease, solitary kidney, or chronic kidney disease. After familiarity with irAE of immunotherapy in different specialties is established, the benefits of immunotherapy will outweigh the disadvantages. Immunotherapy is a two-sided knife for PD-L1-selected bilateral UTUC patients.

## Patient perspective

Her family is glad that the patient is alive and has avoided hemodialysis until now in the first case. However, in the second case, her daughter understood that avoidance of hemodialysis was her mother’s wish, and although we had great oncology outcomes, awful adverse events happened despite us doing our best. Both patient families feel that if this treatment could ensure freedom from hemodialysis, they would like to choose such an innovative treatment strategy.

## Data availability statement

The original contributions presented in the study are included in the article/**Supplementary Material**. Further inquiries can be directed to the corresponding author.

## Ethics statement

The studies involving human participants were reviewed and approved by Chang Gung Medical Foundation- Institutional Review Board (CGMF-IRB) (IRB no. 202100779B0). The patients/participants provided their written informed consent to participate in this study. Written informed consent was obtained from the individual(s) for the publication of any potentially identifiable images or data included in this article.

## Author contributions

W-NC wrote the original draft, constructed the figure, and performed the literature review. Y-CH and D-RH assisted in the literature review and revision of the manuscript. C-SC provided clinical materials and final editing of the manuscript. All authors contributed to the article and approved the submitted version.

## Conflict of interest

The authors declare that the research was conducted in the absence of any commercial or financial relationships that could be construed as a potential conflict of interest.

## Publisher’s note

All claims expressed in this article are solely those of the authors and do not necessarily represent those of their affiliated organizations, or those of the publisher, the editors and the reviewers. Any product that may be evaluated in this article, or claim that may be made by its manufacturer, is not guaranteed or endorsed by the publisher.
