# Supplementary figures and images for: Case Report: Neoadjuvant immunotherapy with pembrolizumab alone for bilateral upper tract urothelial carcinoma is a feasible strategy for kidney sparing and avoidance of hemodialysis
Source: Front Oncol. 2022 Sep 23;12:985177. doi: 10.3389/fonc.2022.985177 (PMC9539752; doi:10.3389/fonc.2022.985177)

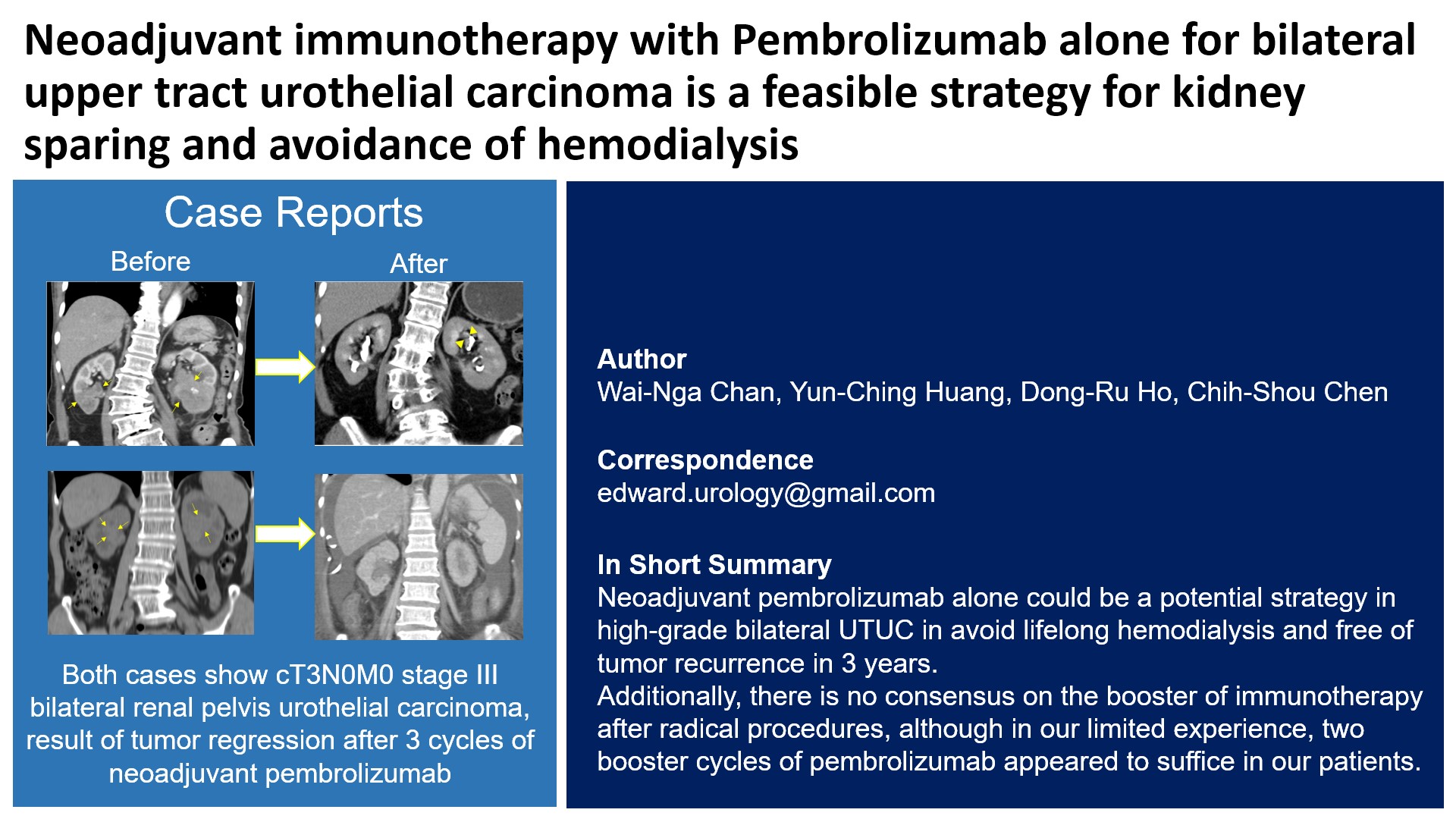

Supplement: Supplementary file 1 [file Image_1.jpeg]
